# Reviving lost binding sites: Exploring calcium‐binding site transitions between human and murine CD23

**DOI:** 10.1002/2211-5463.13214

**Published:** 2021-06-24

**Authors:** Veronica F. Ilkow, Anna M. Davies, Balvinder Dhaliwal, Andrew J. Beavil, Brian J. Sutton, James M. McDonnell

**Affiliations:** ^1^ Randall Centre for Cell & Molecular Biophysics King’s College London UK; ^2^ Asthma UK Centre in Allergic Mechanisms of Asthma London UK

**Keywords:** calcium, CD23, CTLD, FcεRII, IgE, immunoglobulin E

## Abstract

Immunoglobulin E (IgE) is a central regulatory and triggering molecule of allergic immune responses. IgE’s interaction with CD23 modulates both IgE production and functional activities.CD23 is a noncanonical immunoglobulin receptor, unrelated to receptors of other antibody isotypes. Human CD23 is a calcium‐dependent (C‐type) lectin‐like domain that has apparently lost its carbohydrate‐binding capability. The calcium‐binding site classically required for carbohydrate binding in C‐type lectins is absent in human CD23 but is present in the murine molecule. To determine whether the absence of this calcium‐binding site affects the structure and function of human CD23, CD23 mutant proteins with increasingly “murine‐like” sequences were generated. Restoration of the calcium‐binding site was confirmed by NMR spectroscopy, and structures of mutant human CD23 proteins were determined by X‐ray crystallography, although no electron density for calcium was observed. This study offers insights into the evolutionary differences between murine and human CD23 and some of the functional differences between CD23 in different species.

## Graphical abstract

A second calcium‐binding site was engineered into human CD23, based on the amino acid sequence of murine CD23. Chemical shifts from calcium HSQC NMR titrations verified the presence and activity of the engineered site. Differences between murine and human CD23 suggest that modulation of the interaction between CD23 and IgE by calcium may play a greater role in humans.

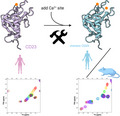

**Abbreviations**
CTLD: C‐type lectin‐like domain
derCD23: a soluble fragment of CD23 cleaved by the house dust mite protease *Der p* 1
Fcε3‐4: subfragment of IgE‐Fc consisting of the dimer of Cε3 and Cε4 domains
IgE: immunoglobulin E
MBL: mannose‐binding lectin
PDB: Protein Data Bank

Allergies are a growing problem, and the prevalence of allergic diseases such as asthma, hay fever and eczema has continued to rise in the industrialised world for more than 50 years [1]. Immunoglobulin E (IgE) is a glycosylated protein belonging to the immunoglobulin family and plays a central role in allergic disease, exerting its effector functions through two receptors: FcεRI and CD23 [2]. FcεRI is primarily expressed on the surface of mast cells and basophils, binds to IgE with high affinity (K_D_ ~ 10^−10^ M) and triggers cellular degranulation after cross‐linking of FcεRI‐bound IgE by allergen [2, 3, 4].

In humans, CD23 is expressed on a range of cells including B cells, T cells, monocytes, follicular dendritic cells, intestinal epithelial cells, bone marrow stromal cells and respiratory epithelial cells. CD23, also referred to as FcεRII, plays a role in a variety of immune functions that include regulation of IgE synthesis, cell survival, cytokine release, antigen presentation, transport of IgE–immune complexes and receptor‐mediated endocytosis [5, 6, 7, 8, 9, 10, 11, 12, 13, 14, 15, 16, 17, 18].

By contrast, CD23 expression in mice is limited to B cells, follicular dendritic cells and enterocytes [14, 19, 20]. CD23 in both humans and mice has two isoforms, CD23a and CD23b, which differ only in their N‐terminal cytoplasmic domain.

CD23 belongs to the C‐type (calcium‐dependent) lectin‐like (CTLD) superfamily of proteins and is a trimer in its membrane‐bound form. A single monomer of CD23 comprises a C‐terminal CTLD globular region connected to a single hydrophobic membrane‐spanning region by an α‐helical coiled‐coil stalk, followed by a short N‐terminal cytoplasmic domain [21, 22]. The stalk region of CD23 is susceptible to proteolysis by proteases such as a disintegrin and metalloproteinase domain‐containing protein 10 (ADAM10) and the major house dust mite protease allergen *Der p* 1 [23, 24]. *Der p* 1 cleaves the trimeric glycoprotein CD23 to release one of the CTLD globular ‘heads’ as a soluble 16 kDa protein, referred to as derCD23 [24, 25].

Although CD23 belongs to the CTLD superfamily, the interaction between IgE and CD23 is carbohydrate independent [26]. A single CD23 domain binds to IgE with low affinity (K_D_ ~ 10^−6^ M) [2, 26]; however, avidity effects mediated by the trimeric form can substantially enhance its affinity for IgE [27, 28].

Membrane CD23 and its soluble fragments have different roles in IgE regulation: as a membrane‐bound protein, CD23 downregulates IgE synthesis upon coligation with membrane IgE by allergen‐IgE complexes, while as a soluble protein, CD23 binds to both membrane IgE and CD21 to upregulate IgE synthesis [2, 28, 29]. Downregulation of IgE synthesis appears to be unique to humans, as in mice CD21 is unable to bind to murine CD23 [22].

Members of the CTLD superfamily, which includes CD23, mannose‐binding lectin (MBL) and DC‐SIGN, are able to bind up to 4 calcium ions, identified by canonical numbering of the four sites (Fig. 1A) [30]. Human CD23 binds one calcium ion, in site 2, with an affinity of ~ 1.5 mm [26, 31]; residues from loop 4 are responsible for calcium coordination at this site (Fig. 1B). The loop 4 residues coordinating the calcium ion in site 2 imply a structural role for this ion, while convergent evolution in CTLDs suggests a carbohydrate coordinating role for the calcium ion in site 1 as demonstrated by the closely related bovine CD23 (Fig. 1C).

**Fig. 1:**
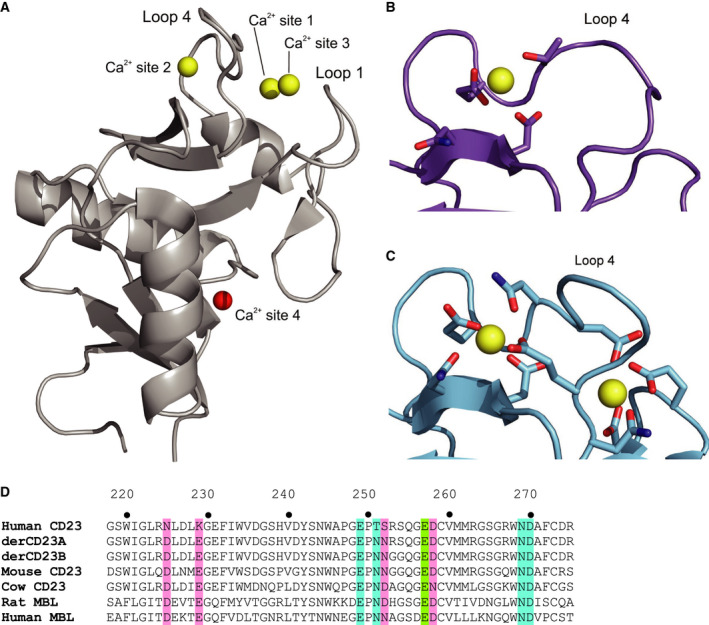
Overall structure of a representative CTLD and the canonical calcium‐binding sites in human and bovine CD23. (A) DC‐SIGN (PDB: 1K9I) [58], shown in grey, is a representative CTLD structure. DC‐SIGN binds three calcium ions, which are shown as yellow spheres. CTLD proteins can bind up to four calcium ions. The location of the fourth site, modelled after superposing the human asialoglycoprotein receptor structure (PDB: 1DV8) [59] onto the DC‐SIGN structure, is shown in red. (B) Calcium‐binding site 2 in human derCD23. Human CD23 binds one calcium ion (PDB: 4G9A) [31]. The structure is rotated 10° about the x‐axis and 35° about the y‐axis relative to the orientation in (A). C) Calcium‐binding sites 1 and 2 in bovine CD23 (PDB: 6PWR) [37]. Bovine CD23 binds two calcium ions. The structure is rotated 10° about the x‐axis and 35° about the y‐axis relative to the orientation in (A). D) A sequence alignment of calcium‐binding sites 1 and 2 in CD23 and MBL. DerCD23^A^ contains the following mutations: Asn225Asp, Lys229Glu, Thr251Asn and Ser252Asn. derCD23^B^ additionally contains the Arg253Gly and Ser254Gly mutations. Residues forming calcium‐binding site 1 are highlighted in pink, and those forming calcium‐binding site 2 are highlighted in blue. Residue Glu257 (highlighted in green) is involved in both calcium‐binding sites. Rat [60] and human MBL [32] both bind a calcium ion at sites 1 and 2.

On the other hand, amino acid sequence comparison of murine CD23 and MBL (Fig. 1D), which binds three calcium ions [32], suggests that murine CD23 can bind up to two calcium ions (sites 1 and 2), mediated by residues in loops 1 and 4 [36]. Loop 1 in human CD23 does not coordinate calcium, but this region makes an important contribution to the interface with IgE [31, 33, 34, 35]. Two calcium ions are coordinated by loops 1 and 4 in the structure of bovine CD23 [37] (Fig. 1C), which shares a similar amino acid sequence to murine CD23 (Fig. 1D). Conformational differences in the loops of bovine CD23 suggest a different manner of interaction with bovine IgE. The second calcium‐binding site in the mouse protein could offer additional opportunities for modulation or regulation of CD23 structure and dynamics [36].

To study the gain/loss of function of calcium binding in human CD23, we created the second calcium‐binding site by introducing calcium‐ligating residues to form site 1. We characterised these human CD23 mutants by NMR spectroscopy, solved their crystal structures and established that the engineered second calcium‐binding site was functional.

## Results and Discussion

### Design of mutant human derCD23 proteins with an engineered calcium‐binding site

To create calcium‐binding site 1 in human derCD23, two mutant proteins, derCD23^A^ and derCD23^B^, were designed after comparing the amino acid sequences of human and murine CD23 (Fig. 1D). Residues in human derCD23 that correspond to aspartic and glutamic acid residues at calcium‐coordinating positions preceding and within loop 1 of murine CD23 were chosen for mutagenesis. The first mutant, derCD23^A^, contained four mutations: Asn225Asp (loop 1), Lys229Glu (loop 1), Thr251Asn (loop 4) and Ser252Asn (loop 4). As murine CD23 contains glycine residues at positions 253 and 254, compared with arginine and serine, respectively, in human CD23, Arg253Gly and Ser254Gly mutations were additionally incorporated into the second mutant, derCD23^B^, to determine whether these substitutions would affect loop mobility and calcium‐binding ability.

### The engineered second calcium‐binding site in human CD23 is functional

**Fig. 2:**
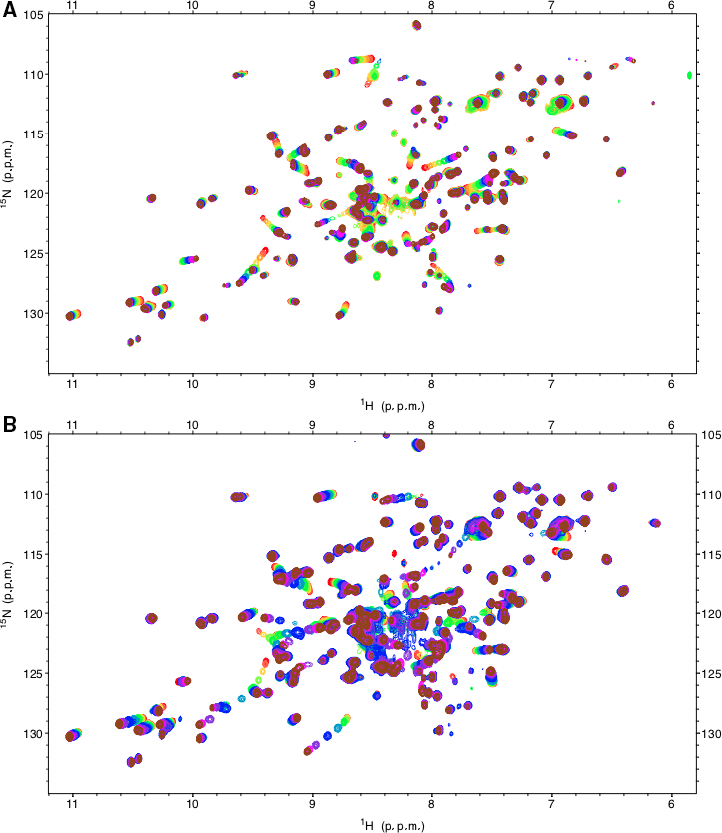
^1^H‐^15^N‐HSQC spectra from a calcium titration into derCD23. (A) WT human derCD23. The colour code for the calcium titrations is as follows: 0 mm (red), 0.1 mm (coral), 0.2 mm (orange), 0.3 mm (gold), 0.4 mm (light green), 0.6 mm (dark green), 1 mm (light blue), 2 mm (dark blue), 4 mm (violet), 10 mm (magenta), 25 mm (brown). The spectra for the 50 mm CaCl_2_ titration were omitted from this figure for clarity. (B) Human derCD23^A^. The colour code for the calcium titrations is as follows: 0 mm (red), 0.1 mm (coral), 0.3 mm (gold), 0.6 mm (light green), 1 mm (dark green), 2 mm (light blue), 4 mm (dark blue), 10 mm (violet), 25 mm (magenta) and 50 mm CaCl_2_ (brown). Peak assignments for WT derCD23 were based on data deposited in the BMRB database [26]. Most residues for derCD23^A^ were assigned using the same peak positions; a small number of residues showed small deviations from the previously published spectra, and these residues were assigned using ^15^N‐filtered TOCSY and NOESY spectra.

**Fig. 3:**
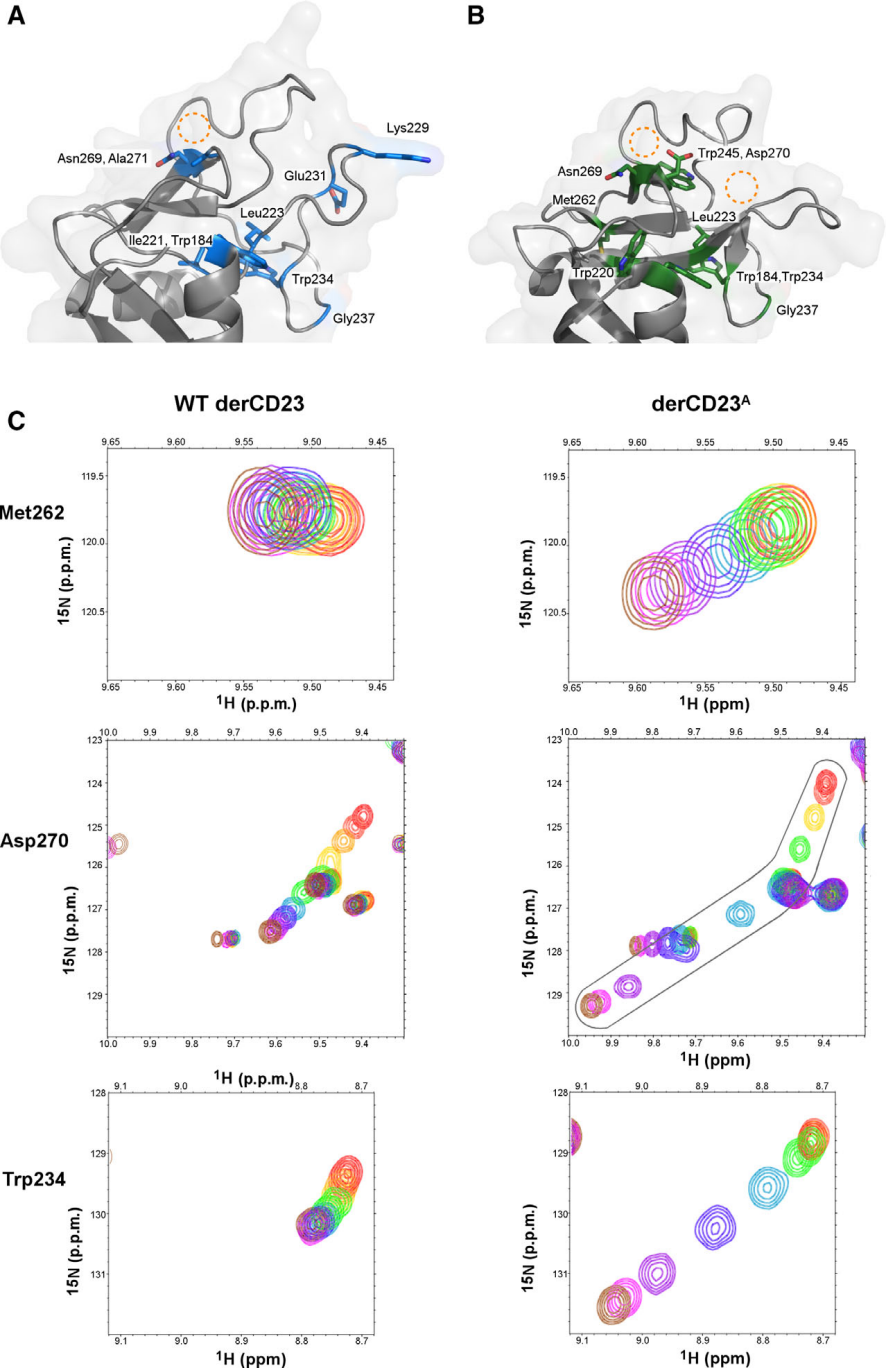
Comparison of residues with different chemical shift perturbations in WT derCD23 and derCD23^A^. (A) A structural representation of residues with large chemical shifts (> 0.08 ppm, blue) located near the calcium‐binding sites of WT derCD23. The expected position of calcium is represented by a dotted circle. (B) A structural representation of residues with chemical shifts (green) with markedly different chemical shifts in derCD23^A^ than in WT derCD23. The expected positions of calcium ions are represented by the dotted circles. Due to mutations in loop 1, not all residues in derCD23^A^ could be unambiguously identified. (C) A comparison of chemical shift changes between WT derCD23 and derCD23^A^ for selected residues (Met262, Asp270 and Trp234). The calcium titration is represented by a rainbow colour code as described in Fig. 2: WT derCD23: 0 mm (red), 0.1 mm (coral), 0.2 mm (orange), 0.3 mm (gold), 0.4 mm (light green), 0.6 mm (dark green), 1 mm (light blue), 2 mm (dark blue), 4 mm (violet), 10 mm (magenta), 25 mm (brown). For derCD23^A^: 0 mm (red), 0.1 mm (coral), 0.3 mm (gold), 0.6 mm (light green), 1 mm (dark green), 2 mm (light blue), 4 mm (dark blue), 10 mm (violet), 25 mm (magenta) and 50 mm CaCl_2_ (brown). The vector represented by Met262 in WT derCD23 changes direction in the titration for derCD23^A^. Asp270 in WT derCD23 follows a linear path, while for Asp270 in derCD23^A^, both the magnitude and direction of chemical shift changes are different. Chemical shift perturbations for the backbone amide of Trp234 are markedly different in WT derCD23 compared with derCD23^A^.

**Fig. 4:**
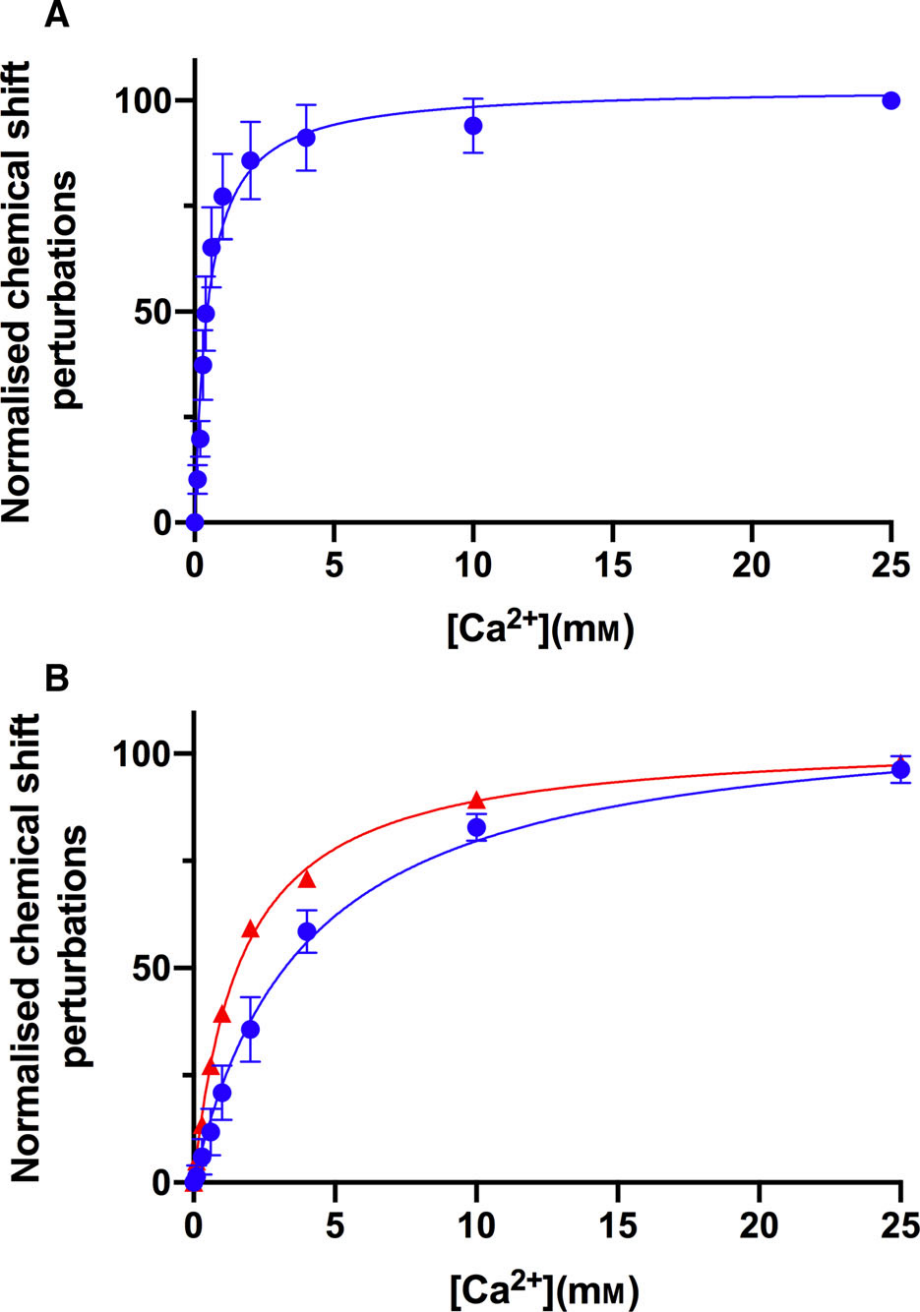
Binding Isotherms for calcium sites in WT derCD23 and derCD23^A^ deduced from the ^1^H‐^15^N‐HSQC spectra calcium titrations. Binding isotherms for WT derCD23 and derCD23^A^ based on the changes in NH chemical shift positions. The chemical shifts have been normalised as described in Section 2.3 to represent the percentage change in the chemical shift perturbations. (A) WT derCD23 has one calcium‐binding site with an apparent K_D_ value of 0.5 ± 0.16 mm. Results and standard deviations are calculated for the averages of residues Trp184 (backbone and sidechain), Ile221 (backbone and sidechain), Leu223, Lys229, Glu231, Trp234, Gly237, Asn269 and Asp270. (B) derCD23^A^ appears to have two distinct calcium‐binding sites (site 1 in red and site 2 in blue). Results and standard deviations are calculated for the averages of residues Trp184 (backbone and sidechain), Ile221 (backbone and sidechain), Leu223, Trp234, Gly237, Met262, Trp268 and Asn269 (all red), and Asp270 (blue). The estimated derCD23^A^ K_D_ values for calcium are 1.7 ± 0.07 mm and 4.0 ± 0.23 mm.

Calcium titrations analysed by ^1^H,^15^N‐HSQC spectra (Fig. 2) revealed several residues with significant chemical shift changes (> 0.08 ppm) (Fig. 3A,B), some of which are responsible for ligating calcium. The WT human derCD23 spectrum (Fig. 2A) was identical to a previously published spectrum, and peak identification was thus performed using the assignment information from the BMRB database [26]. In this titration, the backbone amide of Asp270, a residue that coordinates a calcium ion in site 2, showed a large chemical shift change (> 0.1 ppm). The vector of this change in chemical shift was linear during the addition of calcium (Fig. 3C), and based on the saturation binding curve, the affinity of calcium for this site in WT derCD23 was 0.5 mm (Fig. 4A); this is consistent with the presence of a single calcium‐binding site (site 2) in WT derCD23 and similar to previously described values [31].

The calcium titration for Asp270 in derCD23^A^ showed some marked differences to those observed in WT derCD23 (Fig. 2B). Early points in the calcium titration were linear, and similar to that observed for WT derCD23, but midway through the titration at approximately 2 mm, the direction of the vector changed (Fig. 3C). This change of direction part way through the titration suggested a two‐step process in which one calcium‐binding site, of higher binding affinity, bound calcium at lower concentrations; a second binding site, of lower affinity, was occupied at higher calcium concentrations. Residue Asp270 is uniquely positioned within β–strand 4 and borders both site 1 and site 2, potentially contributing two oxygen atoms to coordinate calcium. This residue can therefore sense calcium binding induced conformational changes at both sites (Fig. S1).

Further analysis revealed that Asp270 was not the only residue that experienced the addition of calcium differently in derCD23^A^ compared with WT derCD23, as illustrated in Fig. 3A,B. The vector for Met262, a residue on the adjacent β‐strand to Asp270, changed direction in derCD23^A^ compared with WT derCD23 (Fig. 3C). The magnitude in chemical shift change for Trp234, situated beneath the calcium‐binding sites, markedly increased in derCD23^A^ (Fig. 3C); this could suggest changes in its hydrogen bonding to Gly237, a residue that also experienced larger chemical shifts in derCD23^A^ compared with WT derCD23. Trp234 is part of a network of aromatic residues that sit beneath calcium‐binding sites 1 and 2; even small changes in the conformation of these aromatic residues are likely to affect chemical shift values of nearby amides due to ring current effects [38]. Apart from Asp270, Met262 and Trp234, greater changes in magnitude were also observed for Trp184, Ile221, Leu223 and Asn269 in derCD23^A^, consistent with these residues being affected by the presence of the second Ca^2+^ ion.

Residues that showed large chemical shifts (> 0.08 ppm) in derCD23^A^ were used to estimate two distinct binding affinities for calcium of approximately 1.7 mm for site 2, and approximately 4 mm for site 1 (Fig. 4B). This suggests that the addition of a new calcium site resulted in a slightly weaker of binding of calcium at site 2, and a restoration of the second calcium‐binding site found in other C‐type lectin‐like domains. Residue Asp270, which sits in loop 4 between sites 1 and 2 (Fig. S1), acts as a reporter residue for both sites, and the observed changes in chemical shift positions during the calcium titration for residue Asp270 indicate a two‐step process for the derCD23^A^ construct with two distinct calcium‐binding affinities.

These observed calcium ion‐binding affinities are comparable to those for other C‐type lectins, which are commonly 0.1–10 mm [39, 40]; this is similar to physiological concentrations of extracellular calcium, which are in the mm range [41]. However, calcium concentrations can rapidly change within the endosome, decreasing by 100‐fold [42]. Sensitivity of CD23 to the calcium concentration could affect ligand processing within the endosome, which might be relevant to the role of CD23 in allergen internalisation and subsequent presentation of these antigens to the immune system [2].

The ^1^H,^15^N‐HSQC spectrum for the derCD23^B^ titration (Fig. S2A) revealed line broadening for residues Arg253‐Glu257 from loop 4, consistent with a change in local dynamics, likely due to the additional Arg253Gly and Ser254Gly mutations. Chemical shift changes for Asp270 (Fig. S2C) therefore could not be analysed for the derCD23^B^ construct in the same way as for derCD23^A^, and it was not possible to determine with complete confidence whether a second calcium‐binding site was present. However, Trp234 (Fig. S2D), located close to the base of site 1, and sensitive to local conformational changes, revealed a larger chemical shift perturbation than in the WT derCD23 calcium titration and showed more similar characteristics to derCD23^A^ than to WT derCD23. The vector for Met262 also changed direction (Fig. S2B) as seen in derCD23^A^ but not WT derCD23.

The calcium titration for derCD23^A^, containing the Asn225Asp, Lys229Glu, Thr251Asn and Ser252Asn mutations, clearly demonstrated that a second, functional calcium‐binding site was successfully engineered into human CD23. These mutant proteins can be used as tools to test how a second calcium may influence CD23 binding to IgE.

### Overall structures of derCD23^A^ and derCD23^B^

**Fig. 5:**
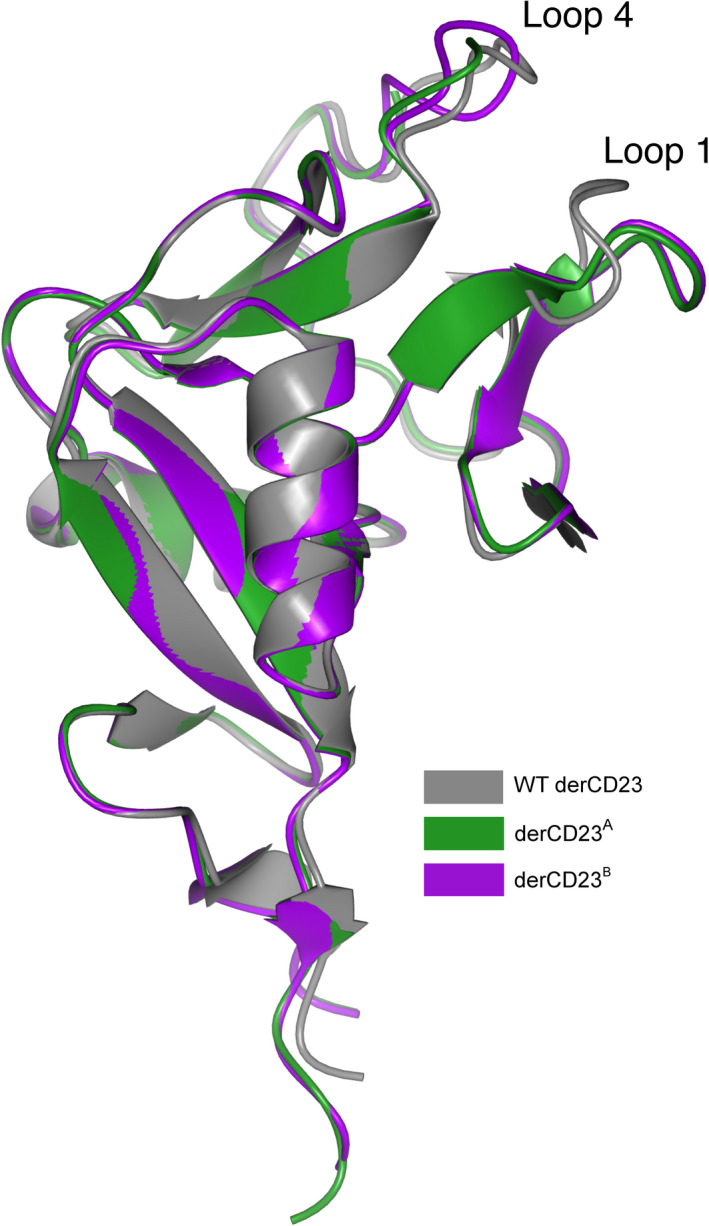
Crystal structures of derCD23^A^ and derCD23^B^. The derCD23^A^ (green, PDB: 6Y0M) and derCD23^B^ (purple, PDB: 6Y0L) structures are superposed on WT human derCD23 (grey) (PDB: 4G9A) [31].

Crystal structures were determined for human derCD23^A^ (containing mutations Asn225Asp, Lys229Glu, Thr251Asn and Ser252Asn) and derCD23^B^ (containing mutations Asn225Asp, Lys229Glu, Thr251Asn, Ser252Asn, Arg253Gly and Ser254Gly) at resolutions of 1.50 Å and 1.65 Å, respectively. The overall root mean square deviation (RMSD) for Cα atoms ranges from 0.42 to 0.92 Å and 0.42 to 0.84 Å for derCD23^A^ and derCD23^B^, respectively, compared with WT derCD23 crystal structures [31, 33, 34, 35, 43] (Fig. 5). Conformational differences in loops 1 and 4 are discussed below.

**Fig. 6:**
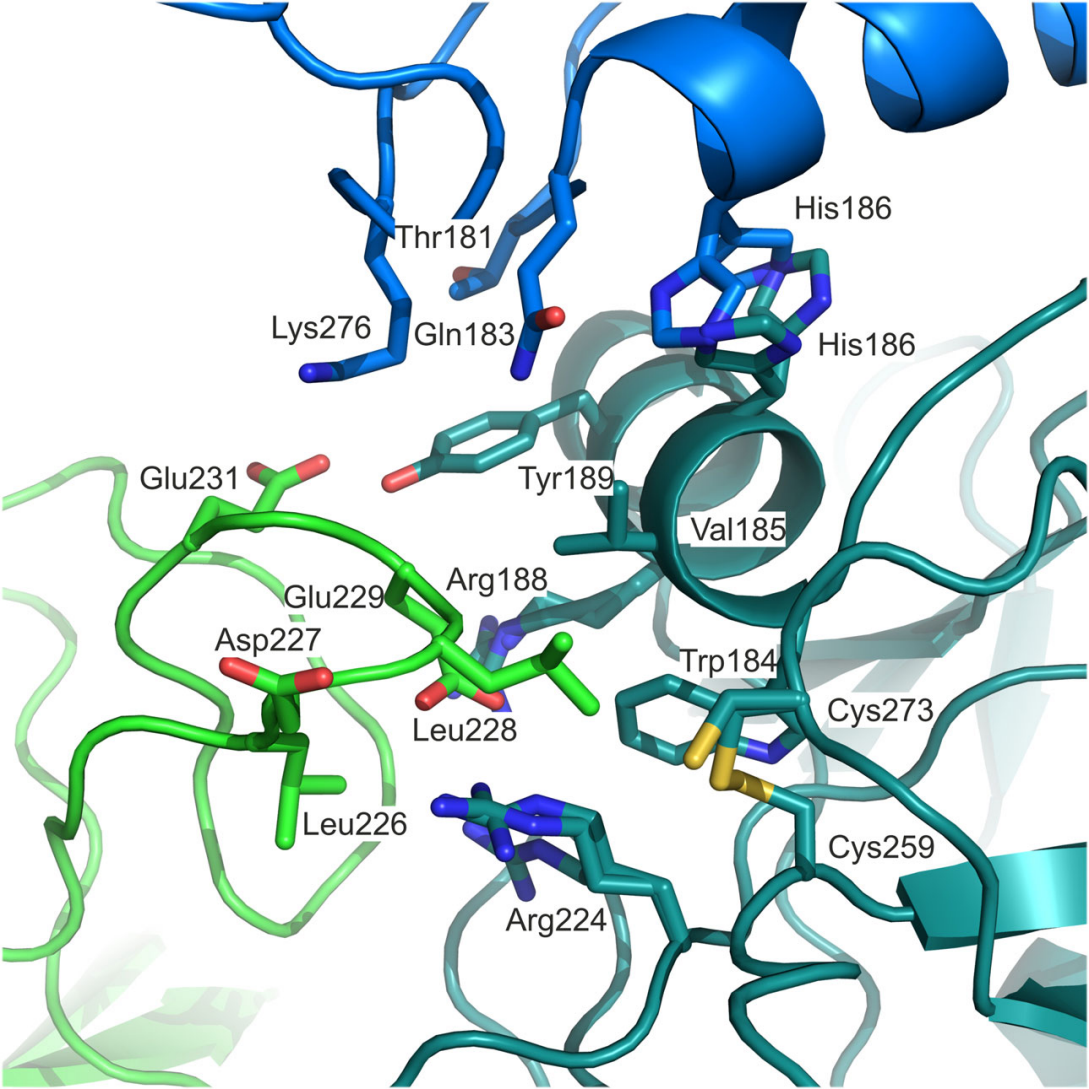
Crystal packing interactions in derCD23^A^. Loop 1 (residues Leu226‐Glu231) in derCD23^A^ (green, PDB: 6Y0M) contacts two symmetry‐related molecules (blue and teal). At this interface, Leu228 contacts Trp184, Val185, Arg224, Cys259 and Cys273 from one symmetry‐related molecule (teal), and Glu229 forms a salt bridge with Arg188.

The derCD23^A^ and derCD23^B^ structures were solved in a new crystal form compared with previously determined CD23 structures [31, 33, 34, 35, 43]. In this new crystal form, loop 1 (residues Leu226‐Glu231) folds away from the protein and contributes to a packing interaction with two symmetry‐related molecules. In the derCD23^A^ structure (Fig. 6), Leu226 contacts Arg224, and Leu228 packs against Trp184, Val185, Arg224, Cys259 and Cys273 from one symmetry‐related molecule. The mutated residue Glu229 (lysine in WT derCD23) contacts Trp184 and Arg224 and forms a salt bridge with Arg188, while Glu231 forms a hydrogen bond with Tyr189. At the interface with a second symmetry‐related molecule, the Leu228 main chain forms a water‐mediated hydrogen bond with Gln183, while the Glu229 main chain forms a hydrogen bond with Lys276. Similar packing interactions are found in the derCD23^B^ structure, but this interface additionally includes a glycerol molecule that forms a hydrogen bond with Glu229.

**Fig. 7:**
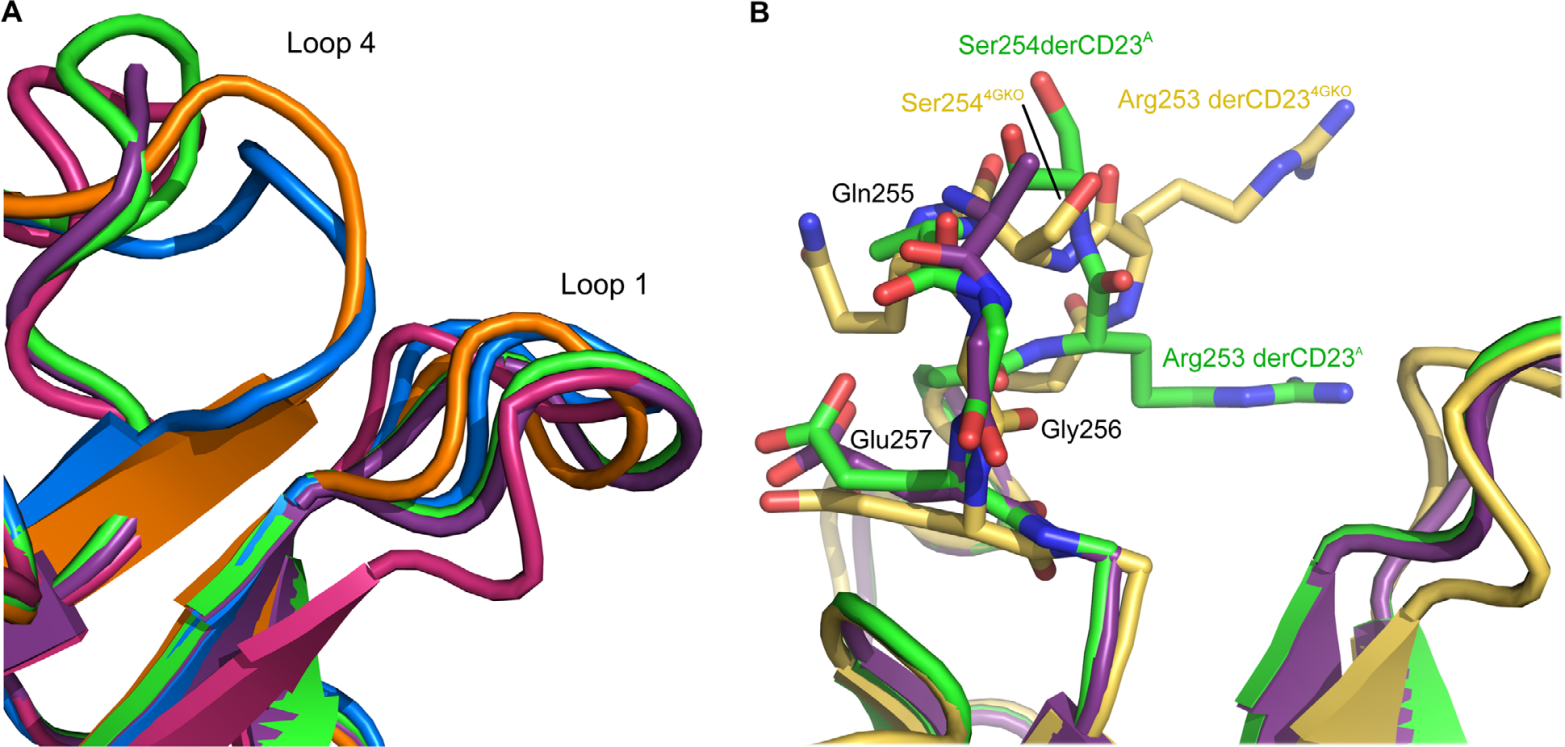
Comparison of loop 1 and loop 4 conformations in derCD23^A^, derCD23^B^ and previous derCD23 crystal forms. (A) The loop 1 conformation in the derCD23^A^ (green) and derCD23^B^ (purple) structures is similar to those in crystal structures of derCD23 in complex with Fcε3‐4 (PDB: 4GKO, pink) [34] and is also found in calcium‐free WT derCD23 (PDB: 4J6Q [33] coloured in blue and PDB 2H2R [43] coloured in orange). (B) The loop 4 conformation in derCD23^A^ (green) and derCD23^B^ (purple) is reminiscent of a conformation found in a structure of calcium‐bound derCD23 in complex with Fcε3‐4 (PDB: 4GKO, yellow) [31], but differs for residues 252–254. Residues with significant differences in sidechain orientations or alignment have been labelled with superscripts; Arg253 and Ser254 were mutated to glycine in derCD23^B^.

Loop 1 contributes to the human IgE‐binding site on CD23 as well as calcium ion coordination in site 1. In crystal structures of CD23 in complex with IgE‐Fc and Fcε3‐4, Leu226, Asp227 and Leu228 from loop 1 contact the IgE Cε3 and Cε4 domains [31, 33, 34, 35]. The loop 1 conformation observed in the derCD23^A^ and derCD23^B^ structures is similar to those observed in the crystal structures of CD23 in complex with IgE‐Fc and Fcε3‐4 (Fig. 7A); this conformation is also found in a crystal structure of calcium‐free WT derCD23 and a Asp270Ala mutant in which loop 1 forms different crystal packing interactions [31, 33, 43].

In human CD23, loop 4 is involved in calcium binding at site 2. Residues Ala246‐Glu257 from this loop are highly flexible in the WT derCD23 NMR structure [26]. A shorter residue range, comprising Thr251‐Glu257, adopts a variety of different conformations in the calcium‐free and calcium‐bound derCD23 crystal structures or is disordered [31, 33, 43]. In the derCD23^A^ structure, backbone atoms for loop 4 residues were modelled, although some side chains were disordered (Fig. 7B). The overall conformation adopted by loop 4 in this structure is reminiscent of the conformation found in a structure of calcium‐bound derCD23 in complex with Fcε3‐4 [31], but differs for residues 252–254; it is possible that the Thr251Asn and Ser252Asn mutations contributed to this conformational difference. By contrast, residues 252–254 were disordered in the derCD23^B^ structure (Fig. 7B). This mutant additionally contains the Arg253Gly and Ser254Gly mutations, which introduce the potential for greater conformational flexibility in the loop.

### Calcium ion‐binding sites

The positions of the residues involved in calcium coordination at site 2 in the derCD23^A^ and derCD23^B^ structures are comparable to those in calcium‐bound human derCD23 structures [31, 43], and the Thr251Asn mutation did not introduce substantial structural changes to the binding site in either mutant.

**Fig. 8:**
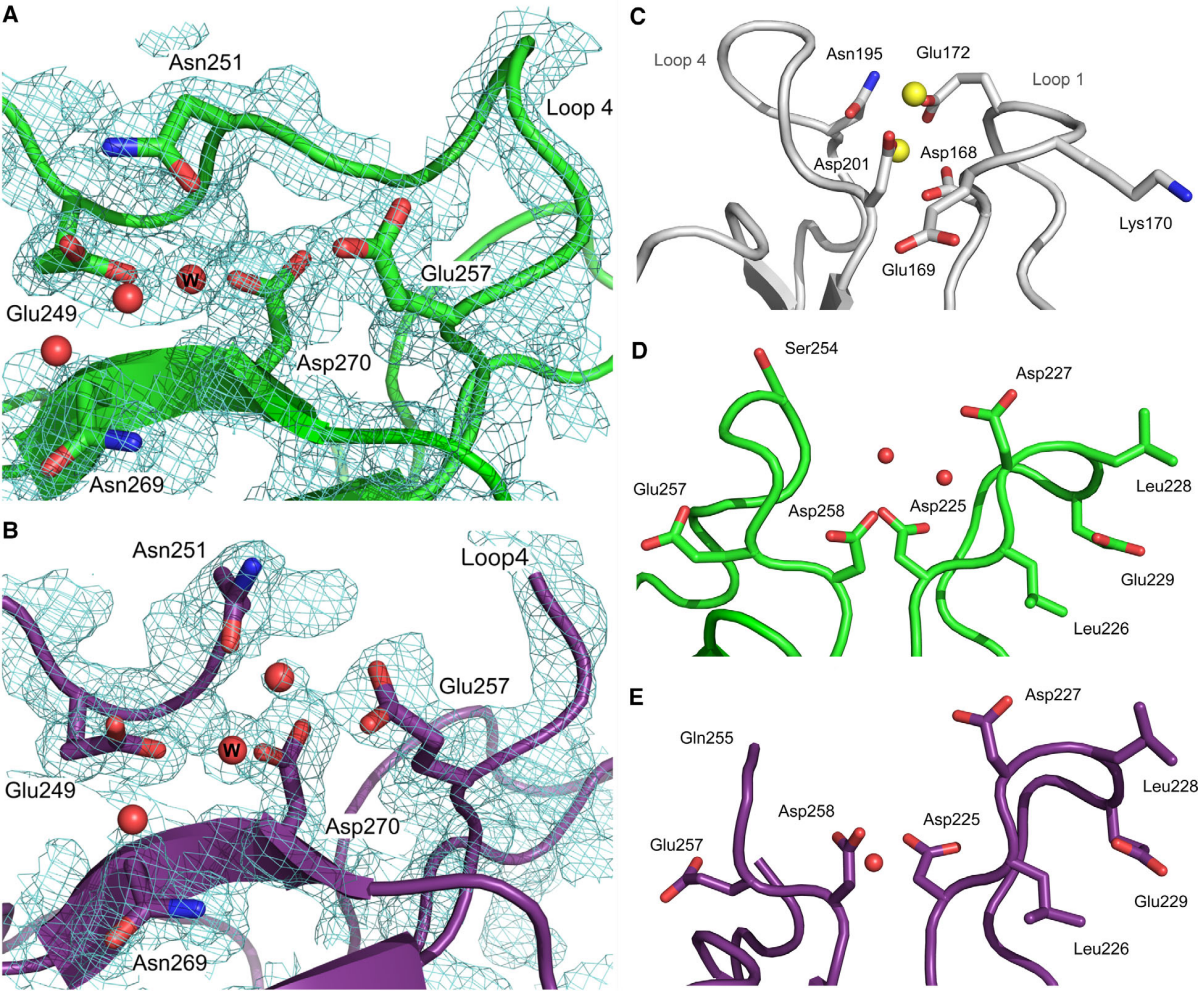
Comparison of calcium‐binding sites 1 and 2 in derCD23^A^, derCD23^B^ and MBL. (A) Crystal structure of derCD23^A^ in the region of calcium‐binding site 2. (B) Crystal structure of derCD23^B^ in the region of calcium‐binding site 2. In both structures, Glu249, Asn251, Glu257, Asn269 and Asp270 are potential calcium‐binding residues, but a water molecule (W) occupies the binding site. A 2F_o_F_c_ electron density map, contoured at 0.9σ, is shown in each panel. (C) MBL calcium‐binding site 1 (PDB: 1HUP) [32]. Two calcium ions, shown as yellow spheres, are bound representing binding to site 1 and the adjacent site 3. (D) derCD23^A^, with two water molecules shown as red spheres. (E) derCD23^B^, with a water molecule shown as a red sphere.

Comparison of the derCD23^A^ structure with crystal structures of calcium‐bound CD23 [31, 43] revealed electron density close to the expected position of a calcium ion at binding site 2 (Fig. 8A). Refinement of a water molecule at this position yielded a *B*‐factor that was comparable with those for nearby protein atoms, but refinement of a calcium ion yielded a *B*‐factor that was approximately twofold higher, and nearby water molecules were displaced. The derCD23^B^ structure likewise revealed electron density close to the expected position of a calcium ion (Fig. 8B). Again, refinement of a water molecule at this position yielded a *B*‐factor that was comparable with those for nearby protein atoms, while refinement of a calcium ion yielded a *B*‐factor that was over twofold higher; furthermore, the coordination sphere for the potential calcium ion was incomplete. Although it is possible that binding site 2 is only partially occupied by calcium in the derCD23^A^ and derCD23^B^ structures, we interpreted the electron density maps as a water molecule at this position; partial occupancy would be consistent with the lower affinity of the mutants for calcium at this site.

Residues in or adjacent to loops 1 and 4 are involved in calcium coordination at site 1. Comparison with residues Asp168 and Asp201 of the MBL structure (Fig. 8C) [32] revealed that the overall positions of two potential calcium‐coordinating residues, Asp225 and Asp258, were not substantially different in the derCD23^A^ and derCD23^B^ structures (Fig. 8D,E), although Asp225 instead adopts a rotamer similar to that found in WT human derCD23. By contrast, the crystal packing interaction in the mutant structures, in which loop 1 folds away from the protein and contacts two symmetry‐related molecules (Fig. 6), would disrupt calcium‐binding site 1 by altering the position of Glu229. Furthermore, the conformation and disorder in loop 4 in the derCD23^A^ and derCD23^B^ structures, respectively, would alter the position of Asn252. Although the NMR‐based calcium titration experiments revealed that a second, functional calcium‐binding site was successfully engineered into derCD23^A^, the electron density maps did not indicate that calcium had bound in this crystal structure.

### Mutant human derCD23 structures provide insights into murine CD23

To date, no crystal structure has been solved for murine derCD23 although crystal structures of bovine CD23, with two calcium ions bound, have recently been reported (Fig. 1C) [37]. The two calcium‐binding sites in bovine CD23 are structurally similar to those in MBL. When the human derCD23 mutant structures were superposed on the bovine CD23 structures (RMSD values ranging from 0.85 to 1.05 Å for Cα atoms), the most significant differences between the structures were the conformations for loops 1 and 4, which are involved in calcium coordination. Calcium‐binding site 2 in bovine CD23 and MBL is structurally similar. In human derCD23, calcium‐binding site 2 exhibits some structural heterogeneity, although the conformations of calcium‐ligating residues Glu249 and Asp270 are similar in the presence of calcium. In the human derCD23 mutants, which both contain Thr251Asn mutations, Asn251 adopts a similar position to the structurally equivalent residues in bovine CD23 and MBL, even in the absence of calcium.

The Asn225Asp and Lys229Glu mutations were designed to render human derCD23 more murine‐like at calcium‐binding site 1. Aspartic and glutamic acid are found at these positions in bovine CD23 (Asp227 and Glu231) and MBL (Asp168 and Glu172) and are involved in calcium coordination in both proteins. Asp225 adopts a similar conformation to that found in WT human derCD23 structures, but this conformation differs from that found in the bovine CD23 and MBL structures in which calcium‐binding site 1 is occupied (Fig. 1). Furthermore, the position of Glu229 in the mutant human derCD23 structures would preclude it from coordinating calcium, as loop 1 adopts a substantially different conformation, forming crystal packing interactions with a symmetry‐related molecule. Nevertheless, the human derCD23 mutant structures described here, together with the bovine CD23 structures, provide a glimpse into how murine CD23 might behave with respect to its interaction with IgE upon calcium binding.

## Conclusions

A calcium‐binding site, conserved in the C‐type lectin‐like domain family, but lost in human CD23, was restored by protein engineering. Analysis of calcium titrations by NMR spectroscopy clearly demonstrated two calcium‐binding sites in one human CD23 mutant. Crystal structures of two mutants were solved to high resolution; however, crystal packing interactions may have perturbed the calcium‐binding sites and calcium ions were not observed in these structures. Differences between human and murine CD23 provide insights into how binding to IgE might be modulated by calcium, which may be relevant to the processing of IgE–allergen complexes in the endosome and allergen presentation to the immune system.

## Materials and methods

### Site‐directed mutagenesis of human derCD23

Mutations Asn225Asp, Lys229Glu, Thr251Asn and Ser252Asn were introduced into recombinant human derCD23 that was previously cloned into a pET5a vector [26], and this mutant was termed derCD23^A^. Another mutant protein, termed derCD23^B^, additionally contained Arg253Gly and Ser254Gly mutations (Fig. 1D). Mutagenesis was performed using the following conditions: 5 μL Phusion® Master Mix, 0.5 μm forward primer, 0.5 μm reverse primer, 20 ng dsDNA template with ddH_2_O added to a final volume of 10 μL, with the following thermal cycling conditions: initial denaturation at 95 °C for 2 min, 18 cycles of 20‐s denaturation at 95 °C, annealing at 60 °C for 10 s and extension at 68 °C for 2.5 min, and a final extension at 68 °C for 5 min. The primers for PCR incorporating the derCD23 mutations were synthesised by Integrated DNA Technologies Inc (Table S1). The mutated pET5a vectors were transformed into BL21(DE3) competent cells according to the manufacturer’s protocol (NEB).

### Protein expression, refolding and purification

Human wild‐type (WT) and mutant derCD23 proteins comprising amino acids Ser156 to Glu298 were expressed in the *Escherichia coli* host strain BL21(DE3) as inclusion bodies. Inclusion bodies were extracted from cell pellets and solubilised, and the protein refolded according to a method described by Taylor *et al*. [44].

^15^N‐labelled derCD23 proteins were expressed in minimal media with the addition of (^15^NH_4_)_2_SO_4_. All derCD23 proteins, both labelled and unlabelled, were purified by hydrophobic interaction chromatography and eluted into 25 mm Tris/HCl pH 7.5 and 2 mm CaCl_2_. Correct folding of the derCD23 proteins was assessed by one‐dimensional ^1^H NMR at 500 MHz (large dispersion and strong signals of methyl groups between 1.0 and −1.0 ppm).

### NMR spectroscopy

NMR spectroscopy was performed on protein samples in a buffer containing 25 mm Tris pH6.8 and 125 mm NaCl, with protein concentrations of 350–400 μm. Data were collected at 35 °C on Bruker spectrometers equipped with CryoProbes operating at 500 and 700 MHz. For chemical shift perturbation experiments, calcium was titrated into samples of ^15^N‐labelled derCD23 mutant proteins, as described previously [26]. NMR data were processed and visualised using Sparky [45]. Chemical shift changes (∆δ) in ^1^H,^15^N‐HSQC spectra were followed until saturation, and dissociation constants (K_D_) were estimated from the titration curves of residues that showed large chemical shift changes (>0.08 ppm). Calcium‐induced shifts were quantitatively analysed by applying a Pythagorean equation weighted by a factor of 0.2 for ^15^N shifts according to the following equation: ∆δ (^1^H, ^15^N) = [{|∆δ(^1^H)|^2^ + 0.2 x |∆δ(^15^N)|^2^}^0.5^].

### Crystallisation

DerCD23^A^ and DerCD23^B^ crystals were grown at 18 °C using the sitting drop vapour diffusion method. DerCD23^A^ was dialysed into 25 mm Tris/HCl pH 7.5, 125 mm NaCl and 0.05% (w/v) sodium azide. Crystals were grown in 0.1 m sodium cacodylate pH 6.5 and 1 m tri‐sodium citrate using a reservoir volume of 100 μL and drops comprising 100 nL protein solution (4 mg·mL^−1^) and 100 nL reservoir solution. The crystals were soaked in a solution of 0.1 m sodium cacodylate pH 7.1, 1.4 m tri‐sodium citrate and 10 mm CaCl_2_ for 4 days before harvesting; the soak solution also served as a cryoprotectant. The crystals were then flash‐cooled in liquid nitrogen. Although cocrystallisation trials were set up with conditions containing CaCl_2_, crystals failed to grow.

derCD23^B^ was buffer exchanged into 25 mm Tris pH 7.5, 125 mm NaCl and 8 mm CaCl_2_ prior to dispensing on the crystallisation plate. Crystals were grown in 0.1 m sodium cacodylate pH 7, 2.0 m ammonium sulphate, 0.2 m sodium chloride and 8 mm CaCl_2_ using a reservoir volume of 100 μL and drops comprising 100 nL protein solution (4 mg·mL^−1^) and 100 nL reservoir solution. The crystals were cryoprotected by soaking in reservoir solution containing an additional 20% (v/v) glycerol followed by flash‐cooling in liquid nitrogen.

### Structure determination, model building and refinement

Data were collected at beamlines I04‐1 (derCD23^A^) and I02 (derCD23^B^) at the Diamond Light Source (Harwell, UK). Data for the derCD23^A^ crystals were integrated with XDS [46] using the Xia2 pipeline [47] and scaled with AIMLESS [48] from the CCP4 suite [49]. Data for the derCD23^B^ crystals were integrated with iMosflm [50, 51]. The structures were solved by molecular replacement with PHASER [52] using protein atoms from PDB entry 4G9A [31] as the search model. The structures were refined using PHENIX [53], and manual model building was performed with COOT [54]. The quality of the models was assessed with MolProbity [55, 56] within PHENIX. Interfaces were analysed with PISA [57]. Data processing and refinement statistics are presented in Table 1. Figures were prepared with pymol (version 1.7.4.3, Schrödinger,​ New York, NY, USA; Schrödinger, 2011).

**Table 1:** Data processing and refinement statistics.

Data processing		
Structure name	derCD23^A^	derCD23^B^
No. of molecules in asymmetric unit	1	1
Space group	P 6 2 2	P 6 2 2
Unit cell dimensions (Å)	*a* = *b* = 115.19 *c* = 45.75	*a* = *b* = 113.7 *c* = 45.70
Resolution (Å)		
Overall (outer shell)	49.88–1.50 (1.53–1.50)	98.48–1.65 (1.73–1.65)
Completeness (%)^a^	99.9 (99.7)	94.36 (99.8)
Multiplicity^a^	39.0 (35.5)	11.5 (5.6)
Mean ((I)/σ(I))^a^	24.7 (1.7)	16.6 (1.6)
R_merge_ ^a^	0.109 (3.184)	0.094 (0.936)
R_pim_ ^a^	0.018 (0.538)	0.028 (0.413)
CC_1/2_ ^a^	0.998 (0.808)	0.999 (0.580)
Wilson *B*‐factor (Å^2^)	19.3	19.1
Refinement		
R_work_/R_free_ (%)^b^	16.84/21.19	16.67/20.51
No. of reflections	29 150	20 443
RMSD		
Bond lengths (Å)	0.013	0.012
Bond angles (°)	1.32	1.25
Coordinate error (Å)	0.18	0.20
No. of atoms		
Protein	1149	1104
Solvent	159	152
Other		23^c^
Ramachandran plot		
Favoured (%)	98.48	99.22
Allowed (%)	1.52	0.78

^a^ Values in parentheses are for the highest resolution shell.

^b^ R_free_ set comprises 5% of reflections.

^c^ Glycerol, sulphate.

## Conflict of interest

The authors declare no conflict of interests.

## Author contributions

VFI designed experiments, performed experiments, analysed data, produced figures and wrote the manuscript. AMD performed experiments, analysed data, produced figures, wrote and revised the manuscript. BD designed and performed experiments. AJB conceived and supervised experiments. BJS supervised and revised the manuscript. JMM supervised and performed experiments, analysed data, wrote and revised the manuscript.

## Supporting information

Fig. S1. The role of Asp270 in calcium binding in derCD23^A^ and derCD23^B^. Asp270 (atoms represented as sticks) is illustrated in this crystal structure with hydrogens added and the calcium ions superimposed from the DC‐SIGN structure (PDB: 1K9I) [58]. Asp270 is in close proximity to the calcium binding sites and is well placed to sense changes at both sites.

Fig. S2. ^1^H‐^15^N‐HSQC spectra from a titration of derCD23^B^ and calcium and selected residues with large chemical shift perturbations. (A) The colour code for the calcium titrations is as follows: 0 mm (red), 0.1 mm (coral), 0.2 mm (orange), 3 mm (gold), 0.4 mm (light green), 0.6 mm (dark green), 0.8 mm (light blue), 1 mm (dark blue), 2 mm (violet), 4 mm (maroon), 10 mm (magenta) and 25 mm CaCl_2_ (brown). (B–D) Individual residues that show larger changes in chemical shift perturbations than WT derCD2. (B) The vector of chemical shift changes observed for Met262 in WT derCD23 changes direction in derCD23^B^. (C) For residue Asp270, the chemical shift perturbation observed for the calcium titration follows a linear path in WT derCD23, while the vector of these changes in derCD23^B^ has two distinct steps. (D) Chemical shift changes for the backbone amide of Trp234 vector in WT derCD23 markedly changes in magnitude compared to Trp234 in derCD23^B^.

Table S1. List of primers used for site‐directed mutagenesis. F = forward primer. R = reverse primer. Primer sequences listed in 5′ to 3′ format.

## Data Availability

The data that support the findings of this study are presented in the main manuscript or in the supplementary material of this article. The structural data that support these findings are openly available in the wwPDB at https://doi.org/10.2210/pdb6Y0M/pdb for derCD23A and https://doi.org/10.2210/pdb6Y0L/pdb for derCD23^B^.
