# Supplementary figures and images for: Reviving lost binding sites: Exploring calcium‐binding site transitions between human and murine CD23
Source: FEBS Open Bio. 2021 Jun 24;11(7):1827–40. doi: 10.1002/2211-5463.13214 (PMC8255853; doi:10.1002/2211-5463.13214)

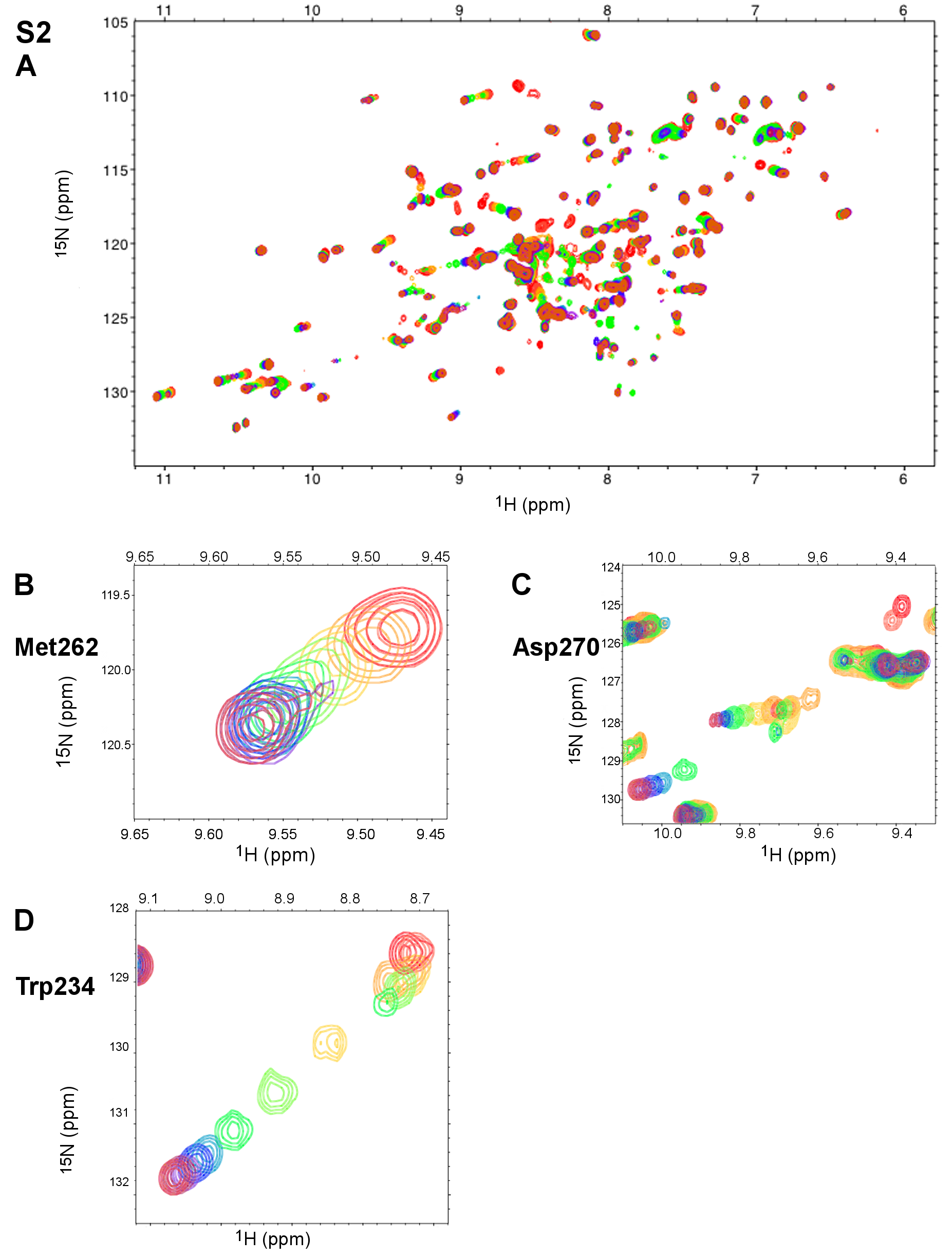

Supplement: Supplementary file 1 — Fig. S1. The role of Asp270 in calcium binding in derCD23A and derCD23B. Asp270 (atoms represented as sticks) is illustrated in this crystal structure with hydrogens added and the calcium ions superimposed from the DC‐SIGN structure (PDB: 1K9I) [58]. Asp270 is in close proximity to the calcium binding sites and is well placed to sense changes at both sites. [file FEB4-11-1827-s001.tif]

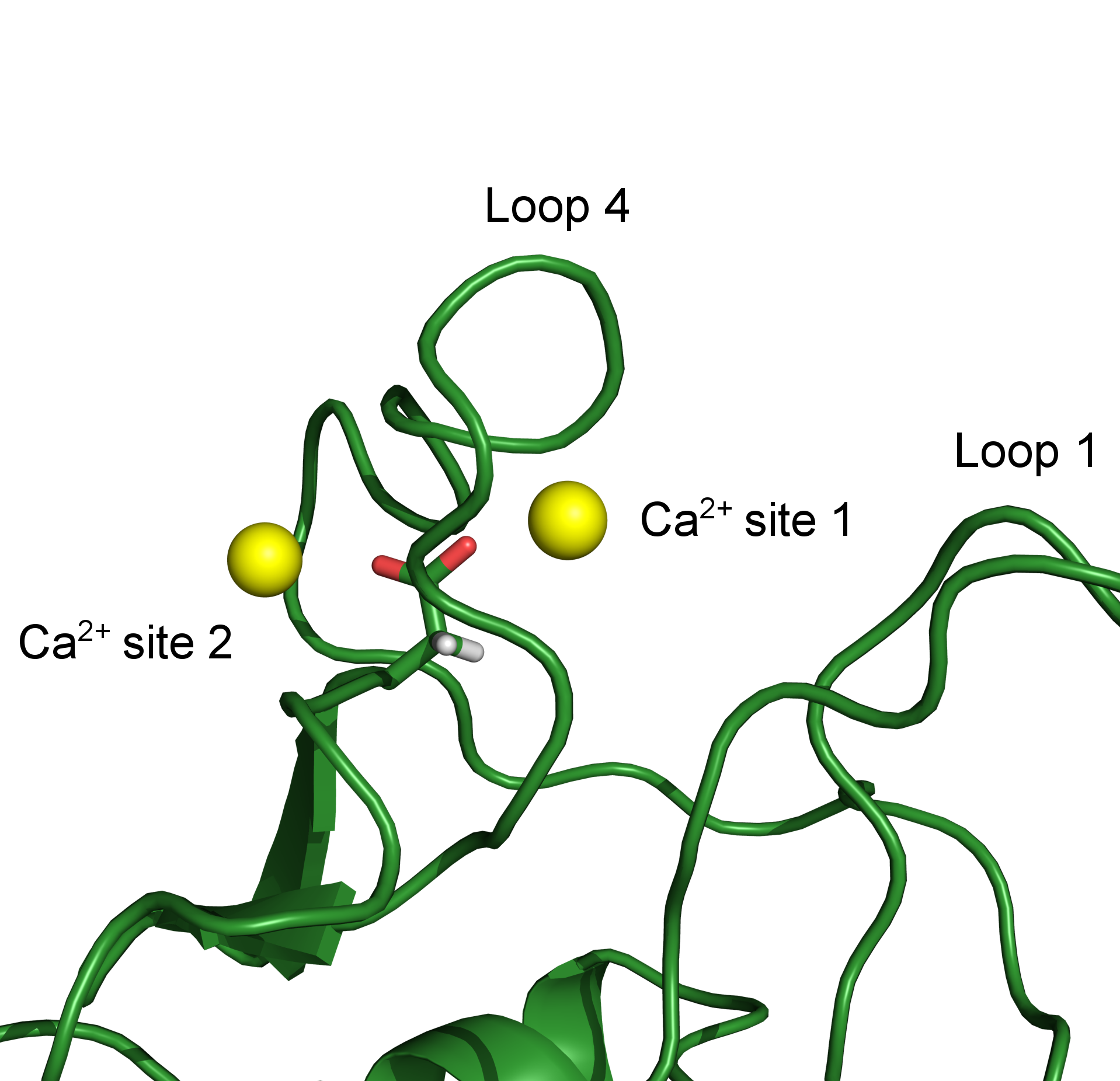

Supplement: Supplementary file 2 — Fig. S2. 1H‐15N‐HSQC spectra from a titration of derCD23B and calcium and selected residues with large chemical shift perturbations. (A) The colour code for the calcium titrations is as follows: 0 mm (red), 0.1 mm (coral), 0.2 mm (orange), 3 mm (gold), 0.4 mm (light green), 0.6 mm (dark green), 0.8 mm (light blue), 1 mm (dark blue), 2 mm (violet), 4 mm (maroon), 10 mm (magenta) and 25 mm CaCl2 (brown). (B–D) Individual residues that show larger changes in chemical shift perturbations than WT derCD2. (B) The vector of chemical shift changes observed for Met262 in WT derCD23 changes direction in derCD23B. (C) For residue Asp270, the chemical shift perturbation observed for the calcium titration follows a linear path in WT derCD23, while the vector of these changes in derCD23B has two distinct steps. (D) Chemical shift changes for the backbone amide of Trp234 vector in WT derCD23 markedly changes in magnitude compared to Trp234 in derCD23B. [file FEB4-11-1827-s003.tif]
