# Supplementary material for: Reviving lost binding sites: Exploring calcium‐binding site transitions between human and murine CD23
Source: FEBS Open Bio. 2021 Jun 24;11(7):1827–40. doi: 10.1002/2211-5463.13214 (PMC8255853; doi:10.1002/2211-5463.13214)
Supplement: Supplementary file 3 — Table S1. List of primers used for site‐directed mutagenesis. F = forward primer. R = reverse primer. Primer sequences listed in 5′ to 3′ format. [file FEB4-11-1827-s002.docx]

**Table S1.**

| N225D F | CCTGGATTGGCCTTCGGGATTTGGACCTGAAGGGAGAG |
| --- | --- |
| N225D R | CTCTCCCTTCAGGTCCAACTACCGAAGGCCAATCCAGGAGCC |
| K229E F | GGCCTTCGGGATTTGGACCTGGAAGGAGAGTTTATCTGGGTGG |
| K229E R | CCACCCAGATAAACTCTCCTTCCAGGTCCAAATCCCGAAGGC |
| S252N F | CTGGGCTCCAGGGGAGCCCACCAACCGGAGCCAGGGCGAGGAC |
| S252N R | AGTCCTCGCCCTGGCTCCGGTTGGTGGGCTCCCCTGGAGCCCAG |
| T251N & S252N F | CTGGGCTCCAGGGGAGCCCAACAACCGGAGCCAGGGCGAGGACTG |
| T251N & S252N R | CAGTCCTCGCCCTGGCTCCGGtTGtTGGGCTCCCCTGGAGCCCAG |
| R253G & S252G F | GGGCTCCAGGGGAGCCCAACAACGGCGGCCAGGGCGAGGACTGC |
| R253G & S252G R | GCAGTCCTCGCCCTGGCCGCCGTTGTTGGGCTCCCCTGGAGCCC |
